# Expanded circulating follicular dendritic cells facilitate immune responses in chronic HBV infection

**DOI:** 10.1186/s12967-020-02584-6

**Published:** 2020-11-07

**Authors:** Xiaoyi Li, Qifan Zhang, Wanyue Zhang, Guofu Ye, Yanchen Ma, Chunhua Wen, Shuqin Gu, Libo Tang, Yongyin Li

**Affiliations:** 1 State Key Laboratory of Organ Failure Research, Guangdong Provincial Key Laboratory of Viral Hepatitis Research, Department of Infectious Diseases, Nanfang Hospital, Southern Medical University, No. 1838 North Guangzhou Avenue, Guangzhou, 510515 China; 2 Division of Hepatobiliopancreatic Surgery, Department of General Surgery, Nanfang Hospital, Southern Medical University, Guangzhou, China; 3 The Air Force Hospital of Southern Theater Command, Guangzhou, China

**Keywords:** Follicular dendritic cells, Hepatitis B virus, B cells, T cells

## Abstract

**Background:**

The restoration of host hepatitis B virus (HBV)-specific antiviral immunity is an effective strategy for hepatitis B recovery. Follicular dendritic cells (FDCs) play a crucial role in immune regulation. The goal of the present study was to investigate the characteristics and functions of FDCs in chronic HBV infection.

**Methods:**

The frequencies of FDCs in peripheral blood, liver, and spleen were measured in patients with chronic HBV infection. Isolated FDCs from splenic tissues of HBV-related liver cirrhosis-induced hypersplenism patients were cultured with autologous intrasplenic CD4^+^ T cells and CD19^+^ B cells.

**Results:**

We observed that patients with chronic HBV infection had a significantly increased frequency of circulating FDCs compared to that of healthy controls. Additionally, the frequency of circulating FDCs was positively correlated with that of intrahepatic and intrasplenic counterparts. Moreover, positive correlations were observed between the frequencies of circulating FDCs and plasmablast and memory B cells, as well as C-X-C motif chemokine receptor type 5 (CXCR5)^+^CD4^+^ T cells and CXCR5^+^CD8^+^ T cells. Notably, in vitro experimental results demonstrated that FDCs derived from splenic tissues of chronic HBV patients facilitated interferon-γ and interleukin-21 production from autologous intrasplenic CD4^+^ T cells and promoted the proliferation of autologous intrasplenic CD19^+^ B cells.

**Conclusions:**

Expanded FDCs in patients with chronic HBV infection may favor host immune responses against HBV. The identification of this unique population of cell may contribute to a better understanding of the immune regulatory mechanisms associated with chronic HBV infection and provide a potential immunotherapeutic target for this disease.

## Background

Chronic hepatitis B virus (HBV) infection is a major global health burden and may lead to progressive liver diseases such as chronic hepatitis, cirrhosis, and hepatocellular carcinoma (HCC). As current antiviral therapies primarily inhibit HBV DNA replication by targeting the process of reverse transcription and have no direct effects on covalently closed circular DNA (cccDNA), chronic hepatitis B (CHB) patients tend to relapse after drug withdrawal [1–4]. Therefore, the restoration of antiviral immunity against HBV may contribute to persistent viral suppression and even achieve durable viral clearance. Antibody against hepatitis B surface antigen (HBsAb) can neutralize circulating hepatitis B surface antigen (HBsAg) and clear HBV particles in vivo [5, 6]. However, the vast majority of patients with chronic HBV infection fail to generate HBsAb, even in patients with the achievement of HBV clearance, suggesting a profoundly compromised humoral immunity in chronic HBV patients. Thus, restoring HBV-specific B cell immunity may be an effective strategy to cure hepatitis B.

Germinal center (GC) is a specialized microstructure that forms in secondary lymphoid tissues following immunization. GC immune response is a critical process during the host-specific immune response. Notably, the immune response of B cells predominantly depends on the GC structure, including clonal expansion, somatic hypermutation, affinity-based selection, and differentiation into plasma cells to produce protective high-affinity antibodies [7, 8]. The initiation and maintenance of GC require the collaboration of multiple leukocytes, including B cells, follicular helper T (Tfh) cells, follicular regulatory T (Tfr) cells, follicular cytotoxic T (Tfc) cells, and follicular dendritic cells (FDCs) [9]. In a previous study, we demonstrated that C-X-C motif chemokine receptor type 5 (CXCR5)^+^ CD4^+^ T cells promote the proliferation of B cells and boost the production of HBV-related antibodies through the mediator interleukin (IL)-21 [10]. We also observed that CXCR5^+^CD8^+^ T cells, a unique population that differs from traditional CD8^+^ T cells, have a greater ability to suppress HBV and facilitate B cells to produce HBV-specific antibodies by secreting IL-21 [11]. However, the characteristics and functions of FDCs in patients with chronic HBV infection remain largely unknown.

A previous study demonstrated that FDCs emerge from perivascular precursors (preFDCs), the expansion of which requires both lymphoid tissue inducer cells and lymphotoxin (LT), and as preFDCs have been confirmed to exist outside lymphoid organs [12–14], we considered that FDCs may exist in peripheral blood [15]. Generally speaking, FDCs are characterized with CD14^+^, CD21^high^, and FDC^+^ (cloned CNA.42), while B cells (CD21^+^CD14^−^), monocytes (CD21^−^CD14^+^) and macrophages (FDC^−^) are excluded in the case of such markers [15]. FDCs retain native antigens in the form of immune complexes (ICs) for prolonged duration and present antigens to B cells during the secondary response. Additionally, FDCs are crucial for the rescue and activation of B cells by secreting IL-6 and B cell-activating factor (BAFF) [17–19]. However, as FDCs are fragile and tightly associated with B cells, it is challenging to perform FDCs isolation and investigate their functions in vitro [17]. In the present study, we preliminarily dissected the characteristics and functions of FDCs in chronic HBV infection.

## Methods

### Patients and samples

Fifty-five treatment-naïve patients with chronic HBV infection were recruited, 31 of whom were classified into immune tolerant carrier (IT; n = 13), hepatitis B e antigen (HBeAg)-positive CHB (n = 9), and inactive carrier (IC; n = 9) groups according to the American Association for the Study of Liver Diseases guidelines [20], and 10 healthy controls (HCs) were also enrolled (Additional file 4: Table S1). Intrahepatic mononuclear cells (HMCs) were collected from 22 treatment-naïve patients with HBV-related HCC who underwent curative hepatectomy, and matched peripheral blood mononuclear cells (PBMCs) were collected from 11 of these individuals. In addition, intrasplenic mononuclear cells (SMCs) were obtained from another 17 patients who underwent splenectomy due to HBV-related liver cirrhosis-induced hypersplenism, with matched PBMCs collected from 11 of these patients (Additional file 5: Table S2). All individuals were recruited at Nanfang Hospital (Guangzhou, China). The exclusion criteria for these studies were coinfection with hepatitis A virus, hepatitis C virus, hepatitis D virus, hepatitis E virus, and human immunodeficiency virus (HIV). Patients with primary biliary cirrhosis and autoimmune diseases were also excluded. All individuals provided written informed consent, and the studies were approved by the Ethical Committee of Nanfang Hospital.

### Mononuclear cells isolation

Twenty milliliters of heparinized blood was collected from patients with chronic HBV infection and HCs. Human HMCs were obtained following a previously described procedure [21]. Briefly, the liver tissues were flushed using 4 °C RPMI-1640 complete medium (Gibco; Thermo Fisher Scientific, Waltham, MA, USA) supplemented with 2% heat-inactivated fetal bovine serum (FBS; Hyclone; GE Healthcare Life Sciences, Logan, UT, USA), 100 µg/mL streptomycin, 100 U/mL penicillin, and 2 mM ethylenediaminetetraacetic acid (EDTA; Ambion; Applied Biosystems, San Mateo, CA, USA). Subsequently, PBMCs and HMCs were isolated by density-gradient centrifugation on Ficoll-Hypaque and cryopreserved in liquid nitrogen. Human SMCs were isolated as previously described [11, 21]. Briefly, the splenic tissue samples (2 cm^3^) were placed into a 70-μm nylon mesh filter in a culture dish with medium, and then the rounded side of a plunger from a 10 mL syringe was used to mechanically crush the tissues. The generated single-cell suspensions were used to obtain SMCs by Ficoll-Hypaque centrifugation. Cell viability, as assessed by trypan blue exclusion, was always higher than 90%.

### Flow cytometry

PBMCs, HMCs, and SMCs were stained with the following monoclonal antibodies (mAbs) for 30 min at 4 °C: CD14-PECy7, CD21-APC, FDC-FITC (cloned CNA.42), CD3-FITC, CD4-PECy7, CD8-APC, CXCR5-Brilliant Violet™ 421, CD19-APC, CD10-PE, CD38-FITC, and CD27-PerCPCy5.5. Dead cells were excluded using Live/Dead (Thermo Fisher Scientific), cells were incubated with human BD Fc Block (5 μL/million cells in 100 μL of FACS buffer) for 10 min at room temperature to block the Fc-receptors. All samples were analyzed on a BD FACS CantoII flow cytometer or Aria III flow cytometer (BD Biosciences). The data were analyzed with FlowJo software (Tree Star).

### Enzyme-linked immunosorbent assay (ELISA)

The plasma levels of IL-6, IL-7, IL-15, BAFF (Invitrogen; Carlsbad, CA, USA), and C-X-C motif chemokine ligand 13 (CXCL13; R&D Systems; Minneapolis, MN, USA) and the concentrations of IL-6, IL-21, interferon (IFN)-γ (Invitrogen), and CXCL13 in the culture supernatants were assessed by commercially available ELISA kits according to the manufacturers’ instructions.

### Isolation and cultivation of FDCs

Primary human FDCs were established as previously described [17]. Briefly, human splenic tissues were placed in petri dishes and washed several times with PBS, and then cut into small pieces. The spleen fragments were then digested with 0.5 mM EDTA and 0.25% of trypsin for 20 min at 37 °C, and the reaction was stopped with cold RPMI-1640 complete medium supplemented with 10% heat-inactivated FBS. Then, the suspension was filtered through gauze and centrifuged. Subsequently, the supernatant was discarded, and the cell pellet was suspended in medium before being centrifuged on Ficoll-Hypaque. The interlayer cells were collected and incubated in culture flasks for 1 h at 37 °C in RPMI-1640 complete medium with 10% heat-inactivated FBS to allow the adherence of macrophages and granulocytes. The supernatant cells were washed and incubated in a fibroblast medium, comprising Opti-MEM reduced serum medium (Gibco; Thermo Fisher Scientific) supplemented with 3% FBS, 100 U/mL penicillin, 50 μg/mL gentamicin, and 1 mmol/L glutamine. After incubating overnight, the adherent cells attached to the flask and lymphocytes in the supernatant were discarded. The fibroblast medium was replaced and changed twice a week. FDCs matured after 2–4 weeks of cultivation.

### Immunofluorescence staining and confocal microscopy

Cultured FDCs were transferred into the confocal petri dishes and washed with PBS and fixed in 4% paraformaldehyde for 10 min at room temperature. Then, the cells were treated with 0.5% Triton X-100 for 5 min and blocked with 1% bovine serum albumin (BSA; Fdbio Science; Hangzhou, China) for 30 min at room temperature. Subsequently, the cells were stained with mouse anti-human FDC monoclonal antibody (CNA.42, 1:300, eBioscience) overnight at 4 °C. Following three washes with PBS, the cells were incubated with the goat anti-mouse antibody (1:400, Jackson ImmunoResearch) for 1 h at room temperature. Then, the cells were transferred to confocal petri dishes and stained with DAPI (Abcam). Images were acquired using a confocal laser scanning microscope (Fluoview FV10i; Olympus, Tokyo, Japan).

### Cell culture and supernatant analysis

Cultured FDCs were stimulated with IL-4 (30 ng/mL), IL-10 (30 ng/mL), IL-21 (30 ng/mL), lipopolysaccharide (LPS, 1 μg/mL), Peg-IFNα-2a (2 μg/mL, Roche, Shanghai, China), lymphotoxin-α1β2 (10 ng/mL), tumor necrosis factor (TNF)-α (10 ng/mL), CPG (5 μg/mL), or PMA (50 ng/mL) for 3 days, respectively. Then, the supernatants were collected, and the levels of IL-6 and CXCL13 were assessed by ELISA. Autologous CD4^+^ T cells and CD19^+^ B cells were sorted from SMCs of patients with chronic HBV infection by FACS and cryopreserved in liquid nitrogen. Purified autologous CD4^+^ T cells were thawed and plated in a 96-well plate that was preseeded with FDCs at an 80:1 ratio (CD4^+^ T cells/FDCs), or with medium only as a control, and co-cultured in the presence of IL-2 (10 ng/mL) and anti-CD3/CD28 (10 μg/mL) for 3 days, the supernatants were collected, and the levels of IL-21 and IFN-γ were assessed by ELISA.

### Proliferation assay

Purified intrasplenic CD19^+^ B cells were thawed and labeled with carboxyfluorescein succinimidyl ester (CFSE; 1.5 mM; Molecular Probes; Eugene, OR) and suspended at 10^6^ cells/mL. Labeled cells were plated in a 96-well plate that pre-seeded with FDCs at an 80:1 ratio (CD19^+^ B cells/FDCs), or with medium only as a control, and co-cultured in the presence of CPG (10 μg/mL) for 7 days. The proliferation rate of B cells is expressed as the percentage of cells that diluted CFSE intensity at least once at the time of harvest.

### Statistical analysis

Data are expressed as either the median (range) or the mean ± SD. Statistical analyses were performed using GraphPad Prism v.8.0.1 (La Jolla, CA, USA). Mann–Whitney U test was used when two groups were compared. Correlations between variables were assessed with the Spearman rank-order correlation coefficient. All statistical analyses were based on two-tailed hypothesis tests with a significance level of P < 0.05.

## Results

### Circulating FDCs and intrahepatic and intrasplenic FDCs frequencies are positively correlated in chronically HBV-infected patients

**Fig. 1:**
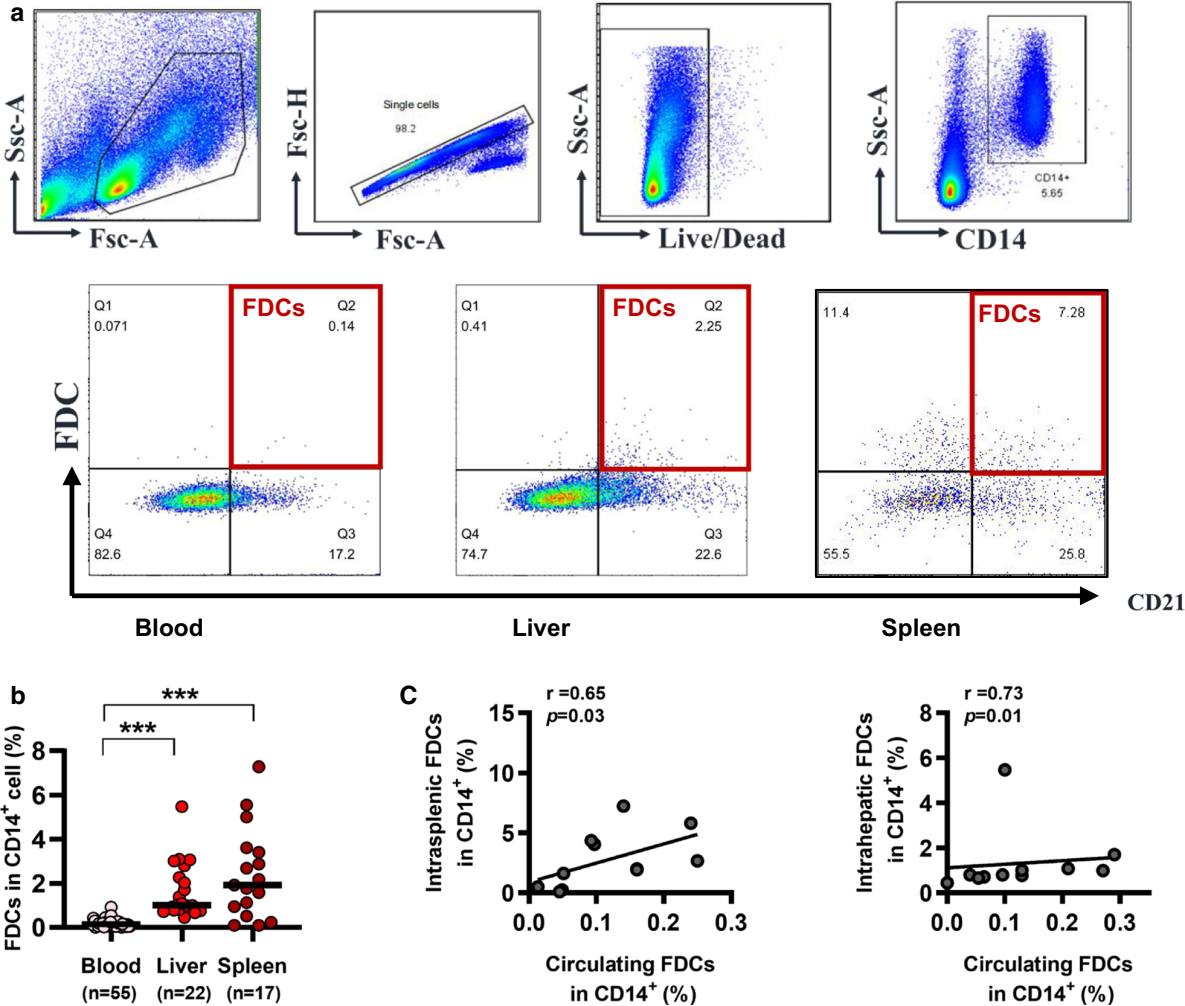
Circulating FDCs and intrahepatic and intrasplenic FDCs frequencies are positively correlated in chronically HBV-infected patients. **a** Gating strategy for the identification of FDCs (CD14^+^CD21^high^FDC^+^) using flow cytometry. FDCs population was calculated as a percentage of CD14^+^ cells. **b** Comparison of the frequencies of circulating (n = 55), intrahepatic (n = 22) and intrasplenic (n = 17) FDCs in patients with chronic HBV infection. **c** The frequency of circulating FDCs was correlated with that of intrasplenic FDCs (left panel, n = 11) and intrahepatic FDCs (right panel, n = 11). ***P < 0.001. FSC-A, forward scatter; SSC-A, side scatter; Live/Dead, fixable dead cell stain

FDCs are primarily located in lymphoid tissue; however, it is difficult to obtain enough lymphoid tissues to investigate the role of FDCs in chronic HBV infection. According to a previous study [16], we quantified the frequencies of FDCs with the indicated gating strategy (Fig. 1a). First, we aim to find out the relationship between intrasplenic and circulating FDCs. Unsurprisingly, the frequency of intrasplenic FDCs was significantly higher than that of circulating FDCs, and a positive correlation between the frequencies of the two cell populations was found (Fig. 1b and c). As hepatitis B virus (HBV) is a prototypical member of the family hepadnaviridae, we then analyzed intrahepatic FDCs. Similar to the intrasplenic counterpart findings, the frequency of intrahepatic FDCs was significantly increased, and a positive correlation between the frequency of intrahepatic FDCs and circulating FDCs was also observed (Fig. 1b and c). Collectively, these findings suggest that analysis of circulating FDCs may serve as an optional surrogate for the assessment of lymphoid FDCs in chronic HBV infection.

### Circulating FDCs are expanded in patients with chronic HBV infection

**Fig. 2:**
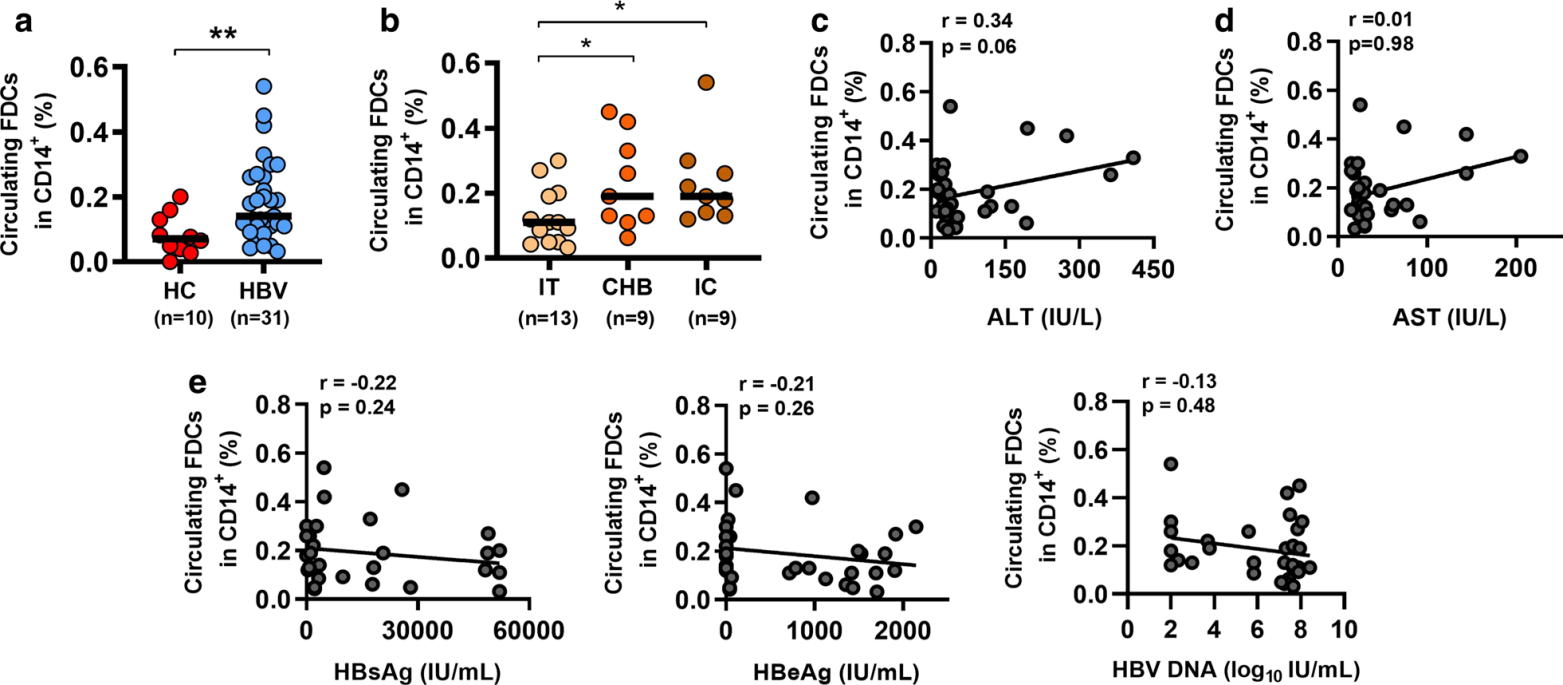
Circulating FDCs are expanded in patients with chronic HBV infection. **a** Comparison of the frequencies of FDCs between healthy controls (HCs, n = 10) and patients with chronic HBV infection (HBV, n = 31). **b** Comparison of the frequencies of FDCs between immune tolerant carrier (IT, n = 13), HBeAg-positive CHB (CHB, n = 9), and inactive carrier (IC, n = 9) patients. **c**–**e** Analyses of the correlations between the frequency of circulating FDCs and serum levels of ALT, AST, HBsAg, HBeAg, and HBV DNA in chronic HBV patients (n = 31). *P < 0.05, **P < 0.005

To determine whether chronic HBV infection can drive FDCs expansion, we measured the frequencies of circulating FDCs in CD14^+^ cells in patients with chronic HBV infection and HCs. As shown in Fig. 2a, a significantly higher frequency of FDCs was observed in patients with chronic HBV infection than in the HCs group. Notably, compared to IT patients, CHB and IC patients had a significantly increased percentage of FDCs (Fig. 2b). The serum levels of alanine aminotransferase (ALT), aspartate aminotransferase (AST), HBsAg, HBeAg, and HBV DNA are known clinical indicators of HBV infection. We then examined the relationships between the frequency of circulating FDCs and the levels of viral and biochemical parameters in chronic HBV patients. However, we observed the serum levels of ALT, AST, HBsAg, HBeAg, and HBV DNA were not correlated with the frequency of circulating FDCs (Fig. 2c–e). We also analyzed correlations between the frequency of circulating FDCs and the levels of the above parameters in the three individual groups of chronic HBV patients. Moreover, we observed that the frequency of circulating FDCs was inversely correlated with the level of ALT in IT patients. In contrast, a positive correlation between the frequency of circulating FDCs and the levels of HBeAg was observed in IT patients (Additional file 1: Figure S1). Overall, these data indicate that chronic HBV infection may induce the expansion of circulating FDCs.

### Circulating FDCs and immune cell subset frequencies are positively correlated in patients with chronic HBV infection

**Fig. 3:**
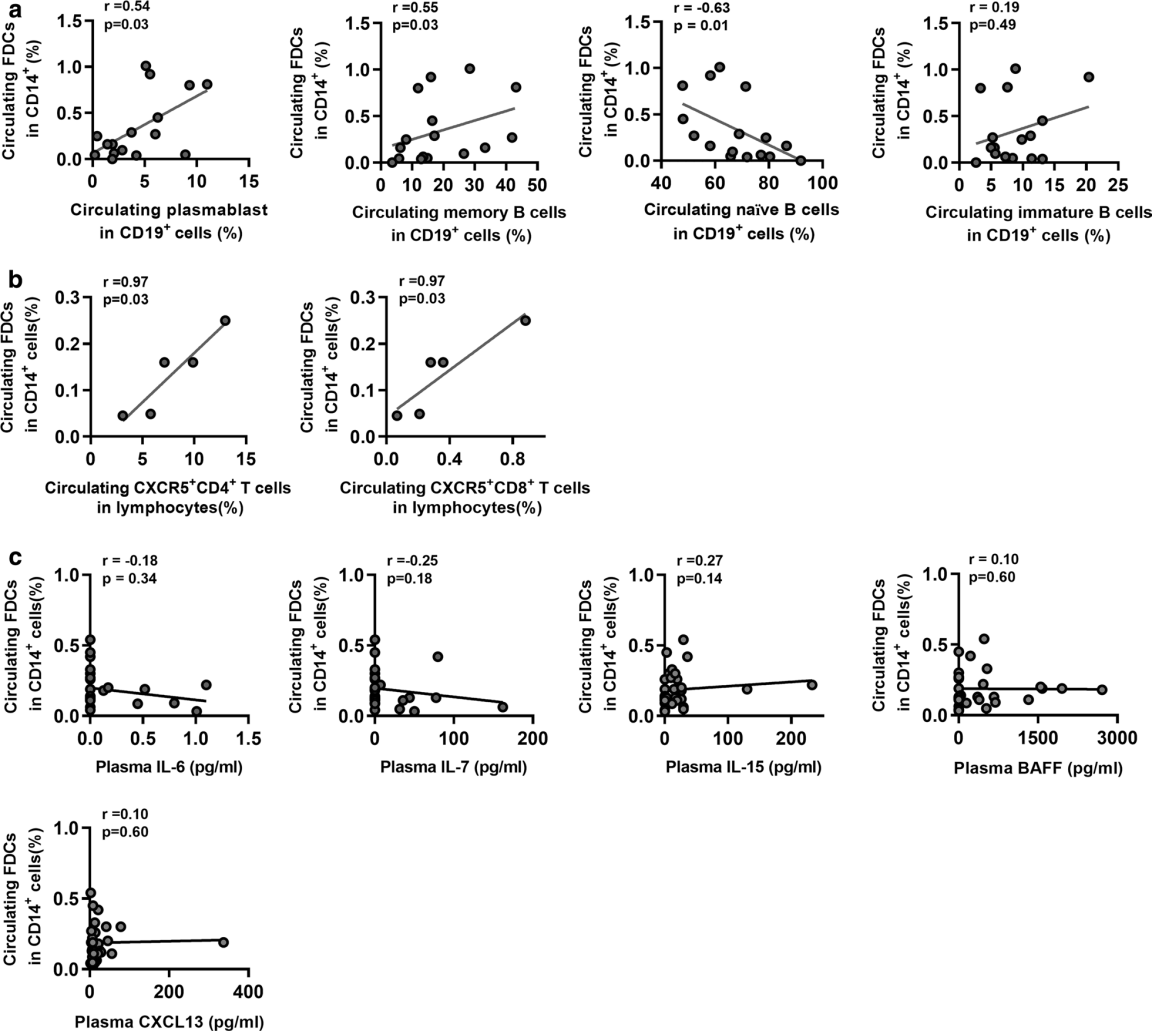
Correlations between the frequencies of FDCs and immune cell subsets in chronic HBV-infected patients. **a** Correlations between the frequencies of FDCs and plasmablast (CD19^+^CD38^+^CD27^+^), memory B cells (CD19^+^CD27^+^CD38^−^), naïve B cells (CD19^+^CD10^−^CD27^−^), and immature B cells (CD19^+^CD10^+^CD27^−^) in peripheral blood in chronic HBV patients (n = 16). **b** Correlations between the frequencies of FDCs and CXCR5^+^CD4^+^ T cell and CXCR5^+^CD8^+^ T cell subsets in peripheral blood in chronic HBV patients (n = 5). **c** Correlations between the frequency of circulating FDCs and plasma levels of IL-6, IL-7, IL-15, BAFF, and CXCL13 in chronic HBV patients (n = 31)

Since FDCs promote B cell proliferation and differentiation by secreting cytokines, such as IL-6, IL-7, IL-15, BAFF, and CXCL13 [19, 22], we subsequently investigated the correlation between the frequencies of circulating FDCs and B cell subsets in patients with chronic HBV infection. Interestingly, we found that the frequency of circulating FDCs was positively correlated with those of plasmablast and memory B cells. In contrast, the frequency of circulating FDCs was inversely correlated with that of circulating naïve B cells, while no significant correlation existed between the frequencies of circulating FDCs and immature B cells (Fig. 3a). We also investigated the relationship between intrasplenic FDCs and intrasplenic B cell subsets. However, no significant correlation was observed between the frequencies of intrasplenic FDCs and B cell subsets (Additional file 2: Figure S2). Moreover, as CXCR5^+^CD4^+^ T cells and CXCR5^+^CD8^+^ T cells are critical for GC immune responses [10, 11], we further investigated the frequencies of these two subpopulations in peripheral blood and the spleen. Importantly, we found that the frequency of circulating FDCs was positively correlated with those of CXCR5^+^CD4^+^ T cells and CXCR5^+^CD8^+^ T cells (Fig. 3b). Correspondingly, similar findings were also observed when comparing intrasplenic FDCs and CXCR5^+^CD4^+^ T cells and CXCR5^+^CD8^+^ T cells (Additional file 3: Figure S3). Additionally, we assessed the plasma levels of IL-6, IL-7, IL-15, BAFF, and CXCL13 in patients with chronic HBV infection by ELISA. However, no significant correlation was found between the frequency of circulating FDCs and plasma levels of the aforementioned cytokines (Fig. 3c). Collectively, these results suggest that FDCs are expanded in chronic HBV infection and may influence B cell differentiation and GC immune responses.

### FDCs facilitate cytokine production from CD4^+^ T cells and the proliferation of B cells

**Fig. 4:**
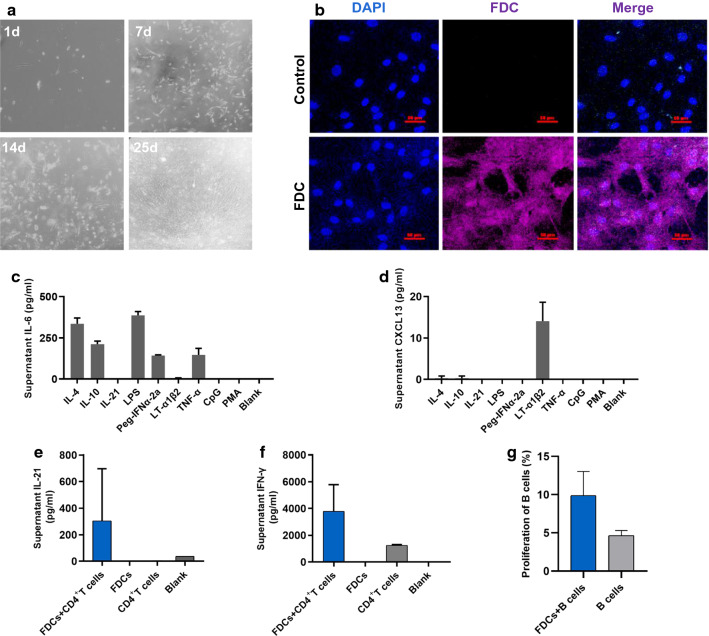
FDCs facilitate CD4^+^ T cell cytokine production and B cell proliferation in vitro. **a** Cultured FDCs were observed by optical microscopy at days 1, 7, 14, and 25. **b** Cultured FDCs were stained for FDC expression and analyzed by confocal microscopy, where red and blue indicate FDCs and DAPI, respectively. Images at ×40 magnification. Cultured FDCs were stimulated with the indicated reagents for 3 days and the supernatants were collected for IL-6 (**c**) and CXCL13 (**d**) detection by ELISA. Cultured FDCs were co-cultured with purified autologous CD4^+^ T cells in the presence of IL-2 (10 ng/mL) and anti-CD3/CD28 (10 μg/mL) for 3 days and the supernatant was collected for IL-21 (**e**) and IFN-γ (**f**) detection by ELISA. **g** Purified autologous CD19^+^ B cells were labeled with CFSE and then co-cultured with cultured FDCs in the presence of CPG (10 μg/mL) for 7 days and the proliferation of B cells was measured by flow cytometry

To investigate the role of FDCs in vitro, FDCs from splenic tissues were isolated from patients who underwent splenectomy due to HBV-related liver cirrhosis-induced hypersplenism and incubated in the fibroblast medium. After 25 days, the FDC network structure was formed and could be observed with an optical microscope (Fig. 4a). The FDCs were then labeled with an anti-FDC fluorescent dye and were further verified by confocal microscopy (Fig. 4b). To investigate the cytokine production abilities of FDCs in vitro, cultured FDCs were stimulated with different reagents (IL-4, IL-10, IL-21, LPS, Peg-IFNα-2a, LT-α1β2, TNF-α, CpG, or PMA). As shown in Fig. 4c, increased IL-6 levels were observed when FDCs were stimulated with IL-4, IL-10, LPS, Peg-IFNα-2a, and TNF-α stimulation. Intriguingly, the production of chemokine CXCL13 from FDCs was only detected under the stimulation of LT-α1β2 (Fig. 4d). Next, we further assessed the effects of FDCs on T cells and B cells. When cultured FDCs were co-cultured with autogenous intrasplenic CD4^+^ T cells, we found elevated expression of IL-21 and IFN-γ in the culture supernatant (Fig. 4e and f). Also, an increase in the proliferation of autogenous intrasplenic CD19^+^ B cells was observed when they were co-cultured with FDCs (Fig. 4g). In summary, these findings indicate that FDCs may facilitate cytokine production from CD4^+^ T cells and promote B cell proliferation.

## Discussion

Humoral immune responses play an essential role in the control of viral infection. However, previous studies have demonstrated a compromised HBsAg-specific B cell response in patients with chronic HBV infection [23, 24], and the reversal of B cell functional impairment is associated with HBsAg seroconversion [25]. Of note, FDCs have been reported to be essential for B cell survival and motivate the process of affinity maturation during GC reaction [26, 27]. The definition of mouse FDCs with FDC-M1 has been generally accepted, while phenotypes of human FDCs remain controversial [28]. According to the report from a previous study, human FDCs were characterized by CD14^+^ CD21^high^ FDC^+^ in the present study. Based on this definition, B cells (CD21^+^ CD14^−^), monocytes (CD21^−^ CD14^+^), and macrophages (FDC^−^) are excluded [16], although this definition is inadequate due to the tissue specificity of FDCs. Therefore, the FDCs described in this study should be defined more accurately as CD14^+^ FDCs [29].

A previous study demonstrated that FDCs are closely associated with the residency and proliferation of tumor cells in follicle-derived lymphomas, even when the infiltration localizes in the bone marrow and non-lymphoid organs [30]. Moreover, another study demonstrated that the precursors of FDCs are perivascular cells and can develop into mature FDCs under certain conditions [12], which indicates that FDCs may not be restricted to the lymphatic organ. However, FDCs have not been well investigated in peripheral blood. In this study, we detected a relatively high number of FDCs in the liver and spleen samples. Interestingly, we also detected a small quantity of FDCs in the peripheral blood in patients with chronic HBV infection. This phenomenon might be attributed to the directional migration of FDCs. In addition, inflammatory factors may induce the maturation of FDC precursors, and enhance the vascular permeability in chronic HBV infection, leading to the entry of mature FDCs into the blood. The fragile characteristics and tight association of FDCs with B cells makes them difficult to investigate; therefore, an alternative method for the assessment of FDCs in lymphoid organs highly desirable. We herein found a close correlation between circulating FDCs and intrahepatic and intrasplenic FDCs, suggesting that analysis of circulating FDCs may be an optional surrogate for the assessment of lymphoid FDCs in chronic HBV infection.

Recent studies have reported the critical role of FDCs in chronic infectious diseases. Importantly, FDCs are a critical HIV reservoir in which FDC-trapped HIV persists for long periods, resulting in the perpetuation and reignition of the disease [31–33]. FDCs also play an essential role in autoimmune disorders, which frequently display follicles with ICs-bearing FDCs, autoreactive GCs, and ongoing affinity maturation [34, 35]. However, the characteristics and functions of FDCs in HBV infection are poorly understood. In the present study, a significantly higher frequency of circulating FDCs was observed in individuals with chronic HBV infection than in the HCs group. However, there was no correlation between the frequency of circulating FDCs and clinical indicators in chronic HBV infection. To study the role of FDCs in the prognosis of HBV infection, we analyzed and found an increased frequency of circulating FDCs in patients in the immune active phase (CHB) compared to IT patients, indicating that the immune activation-induced inflammatory milieu may promote the expansion of FDCs. Nevertheless, a high frequency of FDCs was also found in IC patients, which might be associated with the influence of HBV antigens. We speculated that the production of large amounts of antigens in patients with chronic HBV infection may inhibit the expansion of FDCs. In contrast, a low load of HBV antigens, as is observed in IC patients, may relieve the suppressed status of FDCs. Previous studies have reported that B cells are programmed to undergo apoptosis in the light zone of GC. Only after positive selection by FDCs and Tfh cells, can B cells survive and return to the dark zone for proliferation cycle or differentiate into plasma cells and then leave the GC to participate in humoral immune response [36]. In this study, we showed that the frequency of circulating FDCs is positively correlated with plasmablast and memory B cells but negatively correlated with naïve B cells in patients with chronic HBV infection, suggesting a potential effect of FDCs in the humoral immune response. Furthermore, as a skeletal component of GC, FDCs may play a critical role in regulating the differentiation and function of immune cells in GC, such as Tfh cells, Tfc cells, and B cells. Previous studies from our group have verified the role of CXCR5^+^CD4^+^ T cells and CXCR5^+^CD8^+^ T cells in HBV control. In the present study, we found a positive association between the frequencies of circulating FDCs and CXCR5^+^CD4^+^ T cells and CXCR5^+^CD8^+^ T cells in both the blood and spleen. In addition, a variety of cytokines that produced by FDCs, such as BAFF, IL-6, and IL-15 are involved in the activation and follicular homing of B-cells [37–40]. However, no correlation between the frequency of circulating FDCs and the plasma levels of cytokines was observed in our analysis. This result may be attributed to FDCs primarily exerting their effects in GC, and the plasma cytokine levels reflect the general condition of individuals.

Due to the rare quantity of FDCs, we established primary human FDCs for in vitro experiments. Notably, in the present study experiments on the functions of FDCs were performed after the cultured FDCs were further verified by immunofluorescence analysis under a confocal microscope. FDCs have been reported to produce high levels of IL-6, which is crucial to proinflammatory and immune regulatory cascades [41]. Besides, FDCs also secrete the chemokine CXCL13, which mediates the homing of B cells towards the GC [19]. Our results indicated that FDCs can produce IL-6 and CXCL13 under stimulation in vitro. It was also found that FDCs promoted CD4^+^ T cells cytokine production and B cell proliferation in the co-culture system, but whether this effect is mediated by IL-6 or CXCL13 requires further investigation. FDCs have been reported to deliver antigens to B cells in the form of ICs [19]. Intriguingly, in the present study, we found that the frequency of circulating FDCs is positively correlated with plasmablast and memory B cells in patients with chronic HBV infection, and FDCs promote the proliferation of B cells in vitro. Whether FDCs can deliver HBV antigens to B cells in the form of HBsAg–HBsAb ICs and whether they can restore the immune functions of HBV-specific B cells warrant further study.

It should be noted that the current study has limitations, especially a lack of in-depth functional research on FDCs, which may be partly attributed to the fragile characteristics and the sparse amount of FDCs in individuals with chronic HBV infection.

## Conclusions

In the present study, the characteristics and functions of FDCs were preliminarily investigated, and revealing an expansion of FDCs and their favorable effects on CD4^+^ T cell cytokine production and B cell proliferation in patients with chronic HBV infection, suggesting the regulatory effects of FDCs on anti-HBV immune responses. This study may provide new insights to promote a better understanding of the immune regulatory mechanism of chronic HBV infection (Additional files 4 and 5).

## Supplementary information

**Additional file 1: Figure S1.** Correlations between the frequency of circulating follicular dendritic cells (FDCs) and clinical indicators in chronically HBV-infected patients during different clinical phases.

**Additional file 2: Figure S2.** Correlations between the frequencies of intrasplenic follicular dendritic cells (FDCs) and B cell subsets in patients who underwent splenectomy due to HBV-related liver cirrhosis-induced hypersplenism.

**Additional file 3: Figure S3.** Correlations between the frequencies of intrasplenic follicular dendritic cells (FDCs) and T cell subsets in patients who underwent splenectomy due to HBV-related liver cirrhosis-induced hypersplenism.

**Additional file 4: Table S1.** Clinical characteristics of healthy controls (HCs) and chronically HBV-infected patients classified as immune tolerant carrier (IT), hepatitis B e antigen (HBeAg)-positive CHB (CHB), and inactive carrier (IC).

**Additional file 5: Table S2.** Clinical characteristics of chronically HBV-infected patients, patients with HBV-related hepatocellular carcinoma (HCC) who underwent curative hepatectomy and those who underwent splenectomy due to HBV-related liver cirrhosis-induced hypersplenism.

## Data Availability

The datasets used and/or analysed during the current study are available from the corresponding author on reasonable request.

## Abbreviations

ALT: Alanine aminotransferase
AST: Aspartate aminotransferase
BAFF: B cell-activating factor
BSA: Bovine serum albumin
cccDNA: Covalently closed circular DNA
CFSE: Carboxyfluorescein succinimidyl ester
CHB: Chronic hepatitis B
CXCL13: C-X-C motif chemokine ligand 13
CXCR5: C-X-C motif chemokine receptor type 5
DCs: Dendritic cells
EDTA: Ethylenediaminetetraacetic acid
FBS: Fetal bovine serum
FDCs: Follicular dendritic cells
GC: Germinal center
HBeAg: Hepatitis B e antigen
HBsAb: Antibody against hepatitis B surface antigen
HBsAg: Hepatitis B surface antigen
HBV: Hepatitis B virus
HCC: Hepatocellular carcinoma
HCs: Healthy controls
HIV: Human immunodeficiency virus
HMCs: Intrahepatic mononuclear cells
IC: Inactive carrier
ICs: Immune complexes
IFN: Interferon
IL: Interleukin
IT: Immune tolerant carrier
LPS: Lipopolysaccharide
LT: Lymphotoxin
mAbs: Monoclonal antibodies
PBMCs: Peripheral blood mononuclear cells
SMCs: Splenic mononuclear cells
Tfc: Follicular cytotoxic T cells
Tfh: Follicular helper T cells
Tfr: Follicular regulatory T cells
TNF: Tumor necrosis factor
