# Supplementary material for: Expanded circulating follicular dendritic cells facilitate immune responses in chronic HBV infection
Source: J Transl Med. 2020 Nov 7;18:417. doi: 10.1186/s12967-020-02584-6 (PMC7648402; doi:10.1186/s12967-020-02584-6)
Supplement: Supplementary file 1 — Additional file 1: Figure S1. Correlations between the frequency of circulating follicular dendritic cells (FDCs) and clinical indicators in chronically HBV-infected patients during different clinical phases. [file 12967_2020_2584_MOESM1_ESM.pdf]

Additional file 1

Figure S1

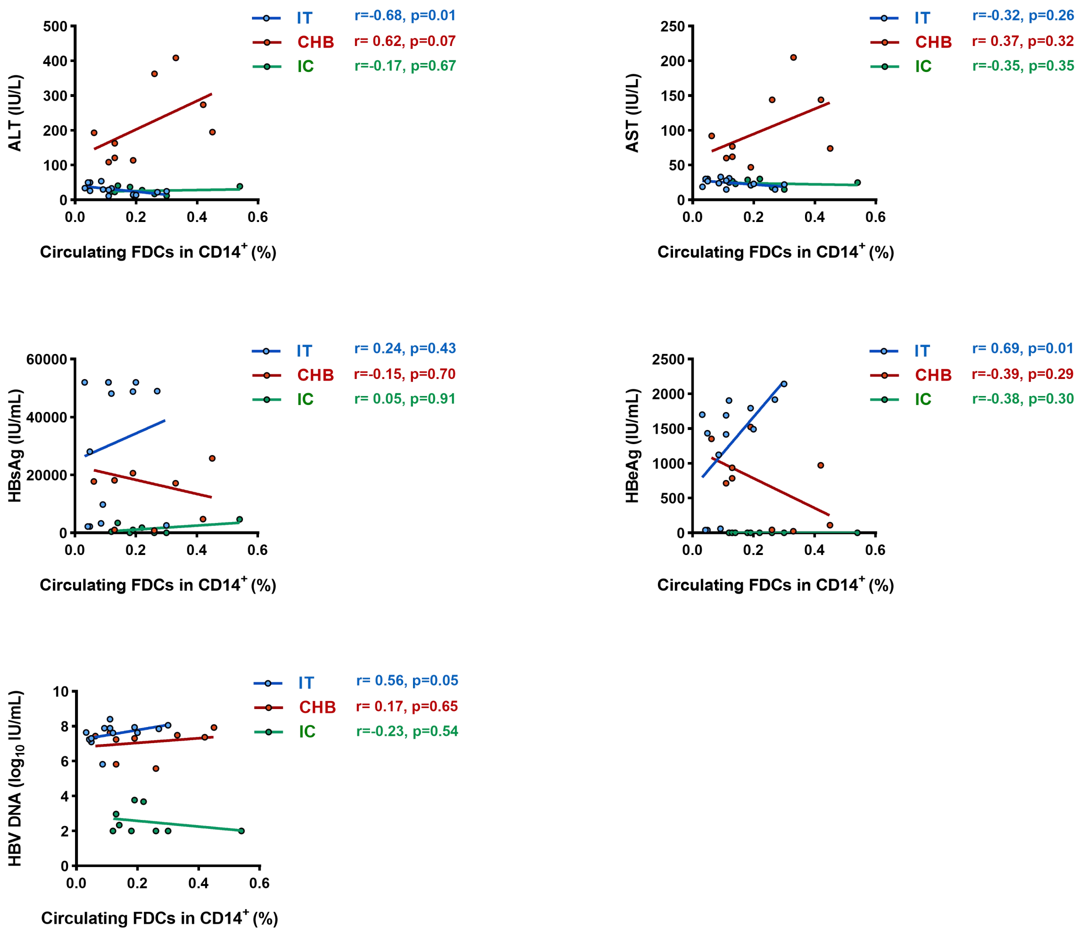

Figure S1. Correlations between the frequency of circulating follicular dendritic cells (FDCs) and clinical indicators in chronically HBV-infected patients during different clinical phases. Correlations between the frequency of circulating FDCs and serum levels of ALT, AST, HBsAg, HBeAg, and HBV DNA in immune tolerant carrier (IT, n = 13), HBeAg-positive CHB (CHB, n = 9), and inactive carrier (IC, n = 9) patients.
