# Supplementary material for: Expanded circulating follicular dendritic cells facilitate immune responses in chronic HBV infection
Source: J Transl Med. 2020 Nov 7;18:417. doi: 10.1186/s12967-020-02584-6 (PMC7648402; doi:10.1186/s12967-020-02584-6)
Supplement: Supplementary file 2 — Additional file 2: Figure S2. Correlations between the frequencies of intrasplenic follicular dendritic cells (FDCs) and B cell subsets in patients who underwent splenectomy due to HBV-related liver cirrhosis-induced hypersplenism. [file 12967_2020_2584_MOESM2_ESM.pdf]

**Additional file 2**

**Figure S2**

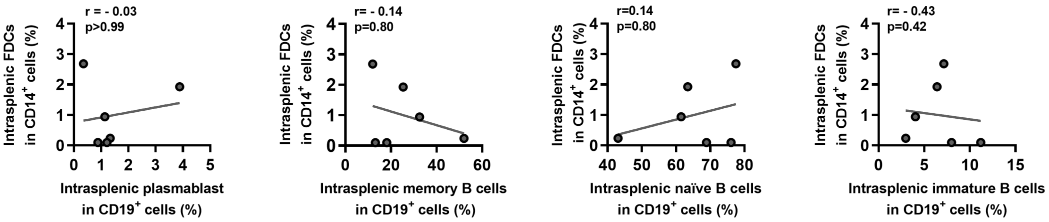


**Figure S2. Correlations between the frequencies of intrasplenic follicular dendritic cells (FDCs) and B cell subsets in patients who underwent splenectomy due to HBV-related liver cirrhosis-induced hypersplenism.** Splenic tissues were obtained from patients who underwent splenectomy due to HBV-related liver cirrhosis-induced hypersplenism (n = 6) and intrasplenic mononuclear cells were isolated, the correlations between the frequencies of intrasplenic follicular dendritic cells (FDCs) and plasmablast ($CD19^+CD38^+CD27^+$), memory B cells ($CD19^+CD27^+CD38^-$), naïve B cells ($CD19^+CD10^-CD27^-$), and immature B cells ($CD19^+CD10^+CD27^-$) were analyzed. Spearman rank correlation test.
