# Supplementary material for: Expanded circulating follicular dendritic cells facilitate immune responses in chronic HBV infection
Source: J Transl Med. 2020 Nov 7;18:417. doi: 10.1186/s12967-020-02584-6 (PMC7648402; doi:10.1186/s12967-020-02584-6)
Supplement: Supplementary file 3 — Additional file 3: Figure S3. Correlations between the frequencies of intrasplenic follicular dendritic cells (FDCs) and T cell subsets in patients who underwent splenectomy due to HBV-related liver cirrhosis-induced hypersplenism. [file 12967_2020_2584_MOESM3_ESM.pdf]

Additional file 3

Figure S3

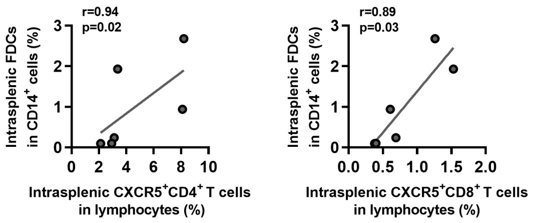

Figure S3. Correlations between the frequencies of intrasplenic follicular dendritic cells (FDCs) and T cell subsets in patients who underwent splenectomy due to HBV-related liver cirrhosis-induced hypersplenism. Splenic tissues were obtained from patients who underwent splenectomy due to HBV-related liver cirrhosis-induced hypersplenism (n = 6) and intrasplenic mononuclear cells were isolated, the correlations between the frequencies of intrasplenic FDCs and CXCR5^{+}CD4^{+} T cells and CXCR5^{+}CD8^{+} T cells were analyzed. Spearman rank correlation test.
