# Supplementary material for: Expanded circulating follicular dendritic cells facilitate immune responses in chronic HBV infection
Source: J Transl Med. 2020 Nov 7;18:417. doi: 10.1186/s12967-020-02584-6 (PMC7648402; doi:10.1186/s12967-020-02584-6)
Supplement: Supplementary file 4 — Additional file 4: Table S1. Clinical characteristics of healthy controls (HCs) and chronically HBV-infected patients classified as immune tolerant carrier (IT), hepatitis B e antigen (HBeAg)-positive CHB (CHB), and inactive carrier (IC). [file 12967_2020_2584_MOESM4_ESM.pdf]

Additional file 4

Table S1. Clinical characteristics of healthy controls (HCs) and chronically HBV-infected patients classified as IT, hepatitis B e antigen (HBeAg)-positive CHB, and IC.

	HCs	IT	CHB	IC
Number	10	13	9	9
Gender (male/female)	6/4	6/7	6/3	6/3
Age (years)*	22 (19-26)	23 (11-57)	45 (20-48)	35 (18-37)
ALT (IU/L)*	NA	29 (11-50)	193 (109-409)	24 (12-41)
AST (IU/L)*	NA	24 (15-33)	77 (47-205)	25 (15-30)
HBV DNA (log_{10} IU/mL)*	NA	7.65 (5.83-8.41)	7.37 (5.58-7.93)	1.98 (1.98-3.78)
HBsAg (IU/mL)*	NA	48142 (2174-52000)	17791 (777.5-52000)	753 (2.47-4672)
HBeAg (positive/negative)	NA	13/0	9/0	0/9

*Data are shown as median (range). ALT, alanine aminotransferase; AST, aspartate aminotransferase; HCs, healthy controls; IT, immune tolerant carrier; CHB, hepatitis B e antigen (HBeAg)-positive chronic hepatitis B; IC, inactive carrier; NA, not applicable. Figure 2a available data from 10 HCs, 13 IT, 9 CHB and 9 IC patients. Figure 2b-e, Figure 3c and Additional file 1: Figure S1 available data from 13 IT, 9 CHB and 9 IC patients.
