# Supplementary material for: Expanded circulating follicular dendritic cells facilitate immune responses in chronic HBV infection
Source: J Transl Med. 2020 Nov 7;18:417. doi: 10.1186/s12967-020-02584-6 (PMC7648402; doi:10.1186/s12967-020-02584-6)
Supplement: Supplementary file 5 — Additional file 5: Table S2. Clinical characteristics of chronically HBV-infected patients, patients with HBV-related hepatocellular carcinoma (HCC) who underwent curative hepatectomy and those who underwent splenectomy due to HBV-related liver cirrhosis-induced hypersplenism. [file 12967_2020_2584_MOESM5_ESM.pdf]

Additional file 5

Table S2. Clinical characteristics of chronically HBV-infected patients, patients with HBV-related hepatocellular carcinoma (HCC) who underwent curative hepatectomy, and patients who underwent splenectomy due to HBV-related liver cirrhosis-induced hypersplenism.

	chronically HBV-infected patients	HBV-related HCC	HBV-related liver cirrhosis -induced hypersplenism
Number	55	22	17
Gender (male/female)	37/18	20/2	14/3
Age (years)*	42 (11-62)	44 (34-62)	43 (34-60)
ALT (IU/L)*	36 (10-409)	32 (10-221)	26 (10-47)
AST (IU/L)*	28 (15-205)	37 (17-146)	26 (15-58)
HBV DNA (log_{10} IU/mL)*	3.79 (1.98-8.41)	3.25 (1.98-7.03)	4.84 (1.98-7.03)
HBsAg (IU/mL)*	1828 (0.23-52000)	248 (0.25-52000)	250 (0.23-250)
HBeAg (positive/negative)	26/29	3/19	3/14

*Data are shown as median (range). ALT, alanine aminotransferase; AST, aspartate aminotransferase. Figure 1b available data from 55 HBV-infected patients, 22 patients with HBV-related HCC and 17 patients with HBV-related liver cirrhosis-induced hypersplenism. Figure 1c available data from 11 patients with HBV-related liver cirrhosis-induced hypersplenism and 11 patients with HBV-related HCC. Figure 3a, available data from 16 HBV-infected patients. Figure 3b, available data from 5 HBV-infected patients.
